# Artificial Neural Network (ANN)-Based Determination of Fractional Contributions from Mixed Fluorophores using Fluorescence Lifetime Measurements

**DOI:** 10.1007/s10895-023-03261-9

**Published:** 2023-05-22

**Authors:** Alexander Netaev, Nicolas Schierbaum, Karsten Seidl

**Affiliations:** 1 Fraunhofer Institute for Microelectronic Circuits and Systems, Finkenstr. 61, 47057 Duisburg, Germany; 2 Department of Electronic Components and Circuits and Center for Nanointegration Duisburg-Essen (CENIDE), University Duisburg-Essen, 47057 Duisburg, Germany

**Keywords:** Fractional contributions, Fluorescence lifetime, Artificial neural networks, Monte Carlo

## Abstract

Here we present an artificial neural network (ANN)-approach to determine the fractional contributions *P*_i_ from fluorophores to a multi-exponential fluorescence decay in time-resolved lifetime measurements. Conventionally, *P*_i_ are determined by extracting two parameters (amplitude and lifetime) for each underlying mono-exponential decay using non-linear fitting. However, in this case parameter estimation is highly sensitive to initial guesses and weighting. In contrast, the ANN-based approach robustly gives the *P*_i_ without knowledge of amplitudes and lifetimes. By experimental measurements and Monte-Carlo simulations, we comprehensively show that accuracy and precision of *P*_i_ determination with ANNs and hence the number of distinguishable fluorophores depend on the fluorescence lifetimes’ differences. For mixtures of up to five fluorophores, we determined the minimum uniform spacing Δ*τ*_min_ between lifetimes to obtain fractional contributions with a standard deviation of 5%. In example, five lifetimes can be distinguished with a respective minimum uniform spacing of approx. 10 ns even when the fluorophores’ emission spectra are overlapping. This study underlines the enormous potential of ANN-based analysis for multi-fluorophore applications in fluorescence lifetime measurements.

**Supplementary Information:**

The online version contains supplementary material available at 10.1007/s10895-023-03261-9.

Fluorophores as labels to visualize proteins, DNA, and other biomolecules have an indispensable role in biomedical applications such as immunohistochemistry [1], imaging microscopy [2], flow cytometry [3], or multiplex assays [4]. When detecting the fluorescence emission of multiple fluorophores in a mixture simultaneously, the emission spectra have to be spectrally separated. One approach is the use of optical filters with different transmission spectra. This also allows then to determine the fractional contributions of each fluorophore based on the intensity in the corresponding transmission bandwidth. In application, due to the issue of optical cross-talk, the maximum number of distinguishable fluorophores and the accuracy of fractional contribution determination is technically limited [5].

An alternative promising approach to distinguish different fluorophores is the measurement of the fluorescence lifetime. Thereby the sample is periodically excited by a short laser pulse and the fluorescence intensity is recorded over time [6]. For most standard fluorophores, the fluorescence intensity decays with a mono-exponential function $I=\alpha {e}^{-t/\tau }$, with an amplitude-representative factor $\alpha$ and the characteristic lifetime $\tau$ [7]. To determine the lifetime, the experimental curve is fitted with this function, where $\alpha$ and $\tau$ are free fit parameters. For a mono-exponential function, conventional non-linear fitting methods work quite stable and in general no constraints to the fit parameters are required to obtain an exact und unique solution for $\alpha$ and $\tau$. [3]

When several fluorophores are simultaneously excited within a mixture (without spatial separation) (Fig. 1a), the fluorescence intensity can follow a multi-exponential decay according to [8] (Fig. 1b)

1 $$I(t)={\sum }_{\mathrm{i}}^{\mathrm{n}}{\alpha }_{\mathrm{i}}{e}^{-\frac{t}{{\tau }_{\mathrm{i}}}},$$

where ${\alpha }_{\mathrm{i}}$ and ${\tau }_{\mathrm{i}}$ are the amplitude-representative factors and the lifetimes of the different fluorophores. These parameters can then be used to calculate the fractional contribution $P$ of each fluorophore to the fluorescence signal of the excited volume [3]

**Fig. 1:**
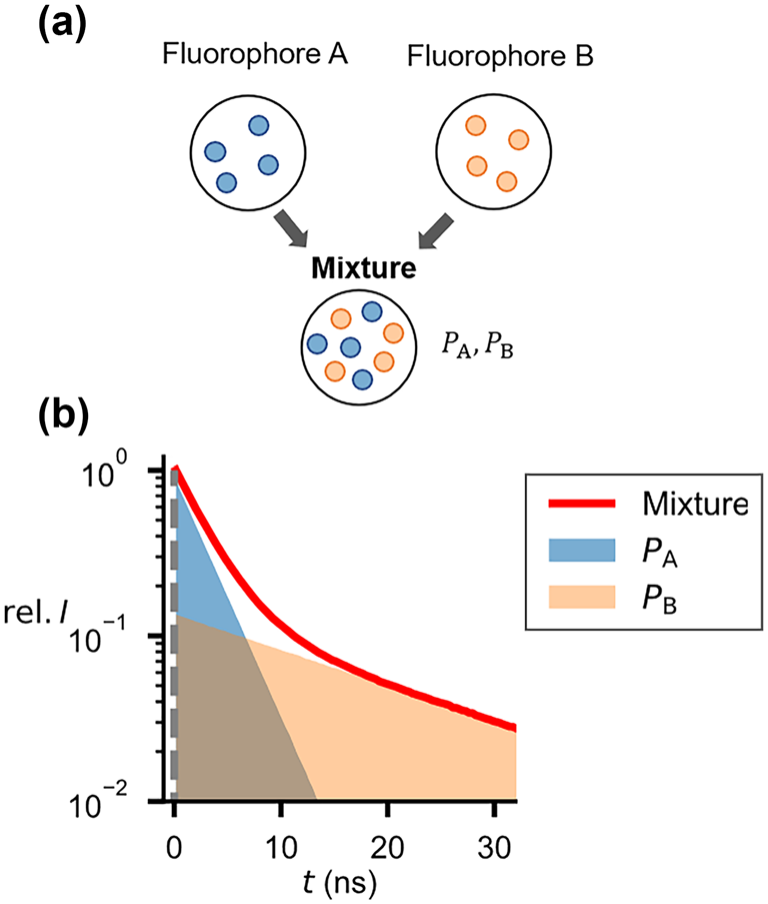
Measurement of fluorescence lifetime in fluorophore mixtures. **a** Mixing two fluorophores A and B with different lifetimes results in a multi-exponential decay of **b** the fluorescence intensity (graph, red curve). The fractional contribution of each fluorophore (${P}_{\mathrm{A}}$, ${P}_{\mathrm{B}}$) to the fluorescence signal is indicated by the light blue and orange area.

2 $$P=\frac{\alpha \tau }{{\sum }_{\mathrm{i}}^{\mathrm{n}}{\alpha }_{\mathrm{i}}{\tau }_{\mathrm{i}}}.$$

Determination of fractional contributions enables quantitative measurement of fluorophore-labeled biomolecules in applications such as biosensing [11], imaging [12], and cytometry [13]. However, these applications require fast algorithms that can extract the fractional contributions in real-time and have the potential to be implemented in data-reducing embedded systems. The most straightforward approach to determine $P$ is to obtain all ${\alpha }_{\mathrm{i}}$ and ${\tau }_{\mathrm{i}}$ by fitting the multiexponential decays. This is an ill-posed problem well-known from literature [9], so there exist multiple solutions for ${\alpha }_{\mathrm{i}}$ and ${\tau }_{\mathrm{i}}$ that well fit to the data. This problem is even more exposed when the amplitude-representative factors ${\alpha }_{\mathrm{i}}$ are disparate and the space between the lifetimes is relatively small which is in general the case in fluorescence lifetime measurements [10]. Applying for example non-linear fitting based on least square (LS) method without constraints can be very instable, which makes it hard to get an exact and unique solution for the fractional contributions [3].

To overcome such fit instability, the fit parameters are typically constrained to certain range of values and fit weighting is optimized [14]. This implies that for example the lifetimes have to be known for precise determination of the amplitude representative factors and hence the fractional contributions. Over the years, several advanced analysis methods have been adapted to analyze multiexponential decays in fluorescence lifetime measurements [15]. With methods such as Bayesian analysis [16, 17] and global method [18, 19] lifetimes and fractional contributions can be determined with high accuracy, but they require large datasets and relatively long computing times [15]. Fit-free methods are either prone to errors from background noise such as phasor approach [20, 21] or not trivial to implement such as Principal Component Analysis (PCA) [22]. Other deconvolution-based methods such as Laplace analysis requires to extrapolate decay data to an arbitrarily infinite time [23–26], while Laguerre expansion show a larger error with shorter and longer lifetimes [20, 27, 28]. Most of those methods have in common that they are relatively complex and require manual optimization at the cost of analysis time. This is even more exposed when lifetimes change by chemical interactions and the fluorescence emission cannot be adequately described by a multiexponential decay function [29].

Here, we propose an alternative approach for automatically determining the fractional contributions from multiexponential decays based on artificial neural networks (ANNs) that does not require prior knowledge and the determination of the lifetimes and therefore no fitting optimization. To our knowledge, this is the first time that ANNs have been used for determination of fractional contributions in fluorescence lifetime measurements. We furthermore evaluated the impact of the lifetimes’ differences between fluorophores within a mixture on the precision and accuracy in the determination of fractional contributions.

To demonstrate the suitability of the ANN-lifetime method for analyzing multiexponential decay functions, we mixed two fluorophores together at different volume ratios (4:1, 2:1, and 1:1 are obtained by mixing three different volumes 25 µl, 50 µl and 100 µl of 2-amino-acridone at 15.6 µM and of acriflavine at 0.2 µM. In total nine different mixing variations) and conducted fluorescence lifetime measurements using a home-built single-photon avalanche diode (SPAD) array-based setup. Each measurement series was repeated three times. The SPAD array (2x192 pixels) is operating in time correlated single-photon counting (TCSPC) mode with a fixed time window of 1.28 µs and a time resolution of 312.5 ps (for technical details, see [6, 30]). The laser pulses and photons collected during the time window were then repeated to obtain fluorescence lifetime data with signal-to-noise ratios (SNR) of at least two orders of magnitude between the maximum intensity and the noise level (see Suppl. Fig. 1). The photon count rate was kept below 2 per measurement window to avoid influence of the detector’s pile-up. As there is an additional delay between each time window of 19.2 µs for data read-out, the effective laser pulse repetition rate is approx. 49 kHz. The repetition rate provides sufficient time for the measurement signal to decay to the noise level [31]. Beside the time resolution of SPAD detector, the minimum lifetime that can be measured is limited by the turn-off time of the laser. Here, we used a laser pulse with FWHM of 1.25 ns and a turn-off time of 600 ps defined as the characteristic lifetime of the laser’s exponential turn-off decay. Since this turn-off time is much smaller than the lifetimes of the here investigated fluorophores, the impact of the laser could be neglected.

The ANN-lifetime method was trained in a first step by Monte-Carlo simulated lifetime curves of fluorophore mixtures at different volume ratios using the lifetimes obtained from a single experiment of each fluorophore. The simulated lifetime curves include characteristics of the SPAD, such as temporal jitter and noise. In a second step, the ANN was trained by experimental fluorescence data of fluorophore mixtures and values of reference (actual) fractional contributions ${P}_{\mathrm{Ref}}$, determined for each mixture by

3 $${P}_{\mathrm{Ref}}=\frac{{I}_{0}v}{{\sum }_{\mathrm{i}}^{\mathrm{n}}{I}_{0,\mathrm{i}}{v}_{\mathrm{i}}},$$

where ${v}_{\mathrm{i}}$ and ${I}_{0}$ are the respective volume fraction and intensity factor. In order to get the intensity factors, a 100 µl sample of each fluorophore was measured separately by intensity-based fluorescence measurement (see Suppl. Fig. 1, 2-amino-acridone: ${\tau }_{1}=10.6 \, \mathrm{ns}$ and acriflavine: ${\tau }_{2}=5.6 \, \mathrm{ns}$). The trained ANN are then applied to experimental curves of fluorophore mixture and outputs the fractional contributions (Fig. 2a). Representative experimental and simulation training curves are shown in Suppl. Figure 3. In this case only the fractional contributions are required for training. However, the ANNs can also be trained on known lifetimes and amplitudes and then the ANN method would also return these parameters.

**Fig. 2:**
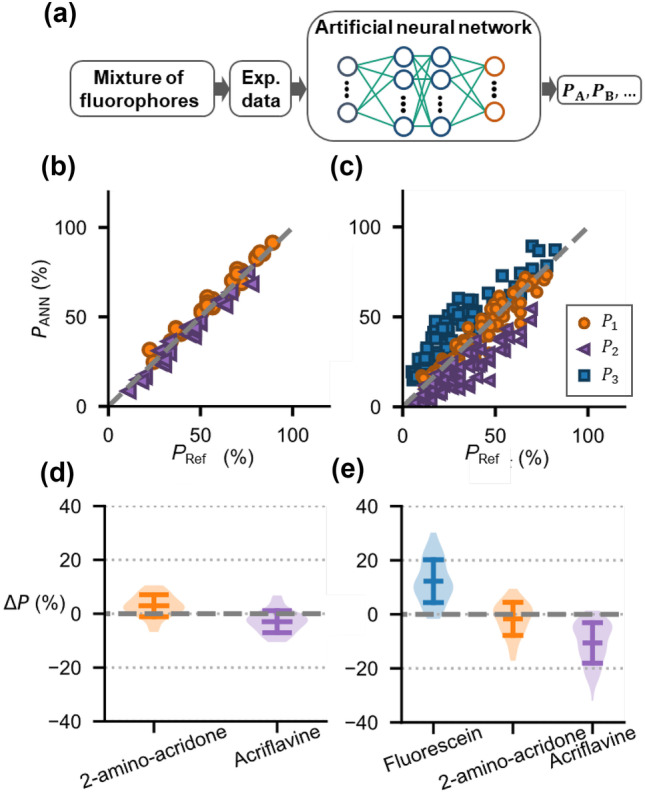
Determination of fractional contributions by artificial neural networks (ANN). **a** Experimental fluorescence lifetime curves of fluorophore mixtures are fed into the ANN that outputs the fractional contributions ${P}_{i}$. (b and c) ${P}_{\mathrm{ANN}}$ vs. ${P}_{ \mathrm{Ref} }$ show that the data points from the mixtures of **b** two fluorophores with 2-amino-acridone (orange circles, ${\tau }_{1}=10.6 \, \mathrm{ns}$) and acriflavine (purple triangles, ${\tau }_{2}=5.6 \, \mathrm{ns}$) and **c** with three fluorophores, additional with fluorescein (blue squares, ${\tau }_{3}=4.1 \, \mathrm{ns}$), are linearly correlated. The gray dashed line corresponds to ${P}_{\mathrm{ANN}}={P}_{\mathrm{Ref}}$. (d and e) Violin plots show the distribution of offsets $\Delta P={P}_{\mathrm{ANN}}-{P}_{\mathrm{Ref}}$ for each fluorophore with respective mean value and standard deviation in mixtures of **d** two and **e** three fluorophores

We used fully connected feed-forward ANN, trained in Python with the PyTorch library [32]. Alternatively, other neural network architectures can be used, such as the Convolutional Neural Network (CNN) [33]. ANN with 3 hidden layers (40, 20, 10 nodes) were used to determine the fractional contributions of two fluorophores, and 4 hidden layers (70, 50, 30, 10 nodes) were used for more than two fluorophores, with a ReLU (rectified linear unit) as activation functions. The number and width of the hidden layers were optimized by trial and error. Backpropagation training integrated in PyTorch with Adam optimization algorithm and mean square error loss function (MSELoss) were used, and the stack size was set to 500 and 20 epochs with a learning rate of 10^-3^.

For the mixture of 2-amino-acridone and acriflavine the determined fractional contributions ${P}_{\mathrm{ANN}}$ by the ANN-lifetime method are highly linear correlated to the set fractional contributions ${P}_{\mathrm{Ref}}$ (${R}^{2}=0.95$) (Fig. 2b).

We then calculated the difference between ${P}_{\mathrm{ANN}}$ to ${P}_{\mathrm{Ref}}$ and the corresponding mean offset

4 $$\langle\Delta P\rangle=\frac1N\cdot\sum P_{\mathrm{ANN}}-P_{\mathrm{Ref}}$$

and its standard deviation

5 $$\upsigma =\sqrt{\frac{1}{N}\cdot \sum {\left|({P}_{\mathrm{ANN}}-{P}_{\mathrm{Ref}})-\langle \Delta P\rangle \right|}^{2}}$$

The mean offset $\langle \Delta P\rangle$ and the standard deviation $\sigma$ thereby provides a measure of accuracy and precision, respectively.

From the measured data in Fig. 2b, we determined $\langle \Delta P\rangle =3.0\%$ and $\sigma =4.1\%$ (Fig. 2d). There is a relative small offset that could be a result of uncertainties in the determination of the reference fractional contributions. Since the ANN-lifetime method directly determines the fractional contributions and not the lifetimes, chemical interactions and non-radiative resonance transfer between the fluorophores that lead to a change in lifetime [3] are compensated by the training process.

Next, we applied the ANN-lifetime method to a mixture of three different fluorophores (Fig. 2c). Therefore, we added fluorescein (at a concentration of 3 µM in different volumes (25 µl, 50 µl, and 100 µl) to the mixtures of 2-amino-acridone and acriflavine, resulting in 27 different mixing variations. Different indices are used here to designate the variables for the experimental data (1 to 3) and for simulated data (A to C).

Figure 2c shows that, for all three fluorophores in the mixtures, ${P}_{\mathrm{ANN}}$ are linearly correlated with ${P}_{\mathrm{Ref}}$ (fluorescein ${R}^{2}=0.65$; 2-amino-acridone ${R}^{2}=0.80;$ acriflavine ${R}^{2}=0.64$). For the mean offset $\langle \Delta P\rangle$ and the standard deviation, we obtained for fluorescein $\langle \Delta P\rangle =7.6\%$ and $\sigma =12.1\%$, for 2-amino-acridone $\langle \Delta P\rangle =0.3\%$ and $\sigma =12.1\%$, and for acriflavine $\langle \Delta P\rangle =-7.9\%$ and $\sigma =10.3\%$ (Fig. 2c), respectively. To compare our results, we also analyzed the measurements using LS method, where we hold the lifetimes fixed during fitting (see Suppl. Fig. 2). In comparison, the ANN-lifetime method determines the fractional contributions with higher precision and accuracy and that in much shorter computation time (factor 1000) than the LS method.

When comparing the obtained values for mixtures of two and three fluorophores, it is noticeable that the linear correlation between ${P}_{\mathrm{ANN}}$ and ${P}_{\mathrm{Ref}}$ is weaker (comparing the ${R}^{2}$-values) and also the mean offsets and standard deviations are higher (Fig. 2d, e). We assumed, that this is due to the larger spacing between the lifetimes in the example of two ($\Delta \tau =5 \, \mathrm{ns}$) in comparison to example of three fluorophores ($\Delta {\tau }_{2-3}=1.5 \, \mathrm{ns};\Delta {\tau }_{1-2}=4.4 \, \mathrm{ns}$), which results in a more precisely determination of the parameters by the ANN-lifetime method.

To underline our assumption, we investigate the influence of the lifetime spacing on the accuracy (mean offset), the precision (standard deviation) and linear correlation (${R}^{2}$) of the determined fractional contributions (Fig. 3). For this purpose, the ANN were trained and tested with a large number ($n={10}^{4}$) of simulated data. We used simulated data since the lifetimes were better adjustable than in experimental settings using real fluorophores. However, it is also possible to adjust the lifetime of certain fluorophores, e.g., with quenching gas [34].

**Fig. 3:**
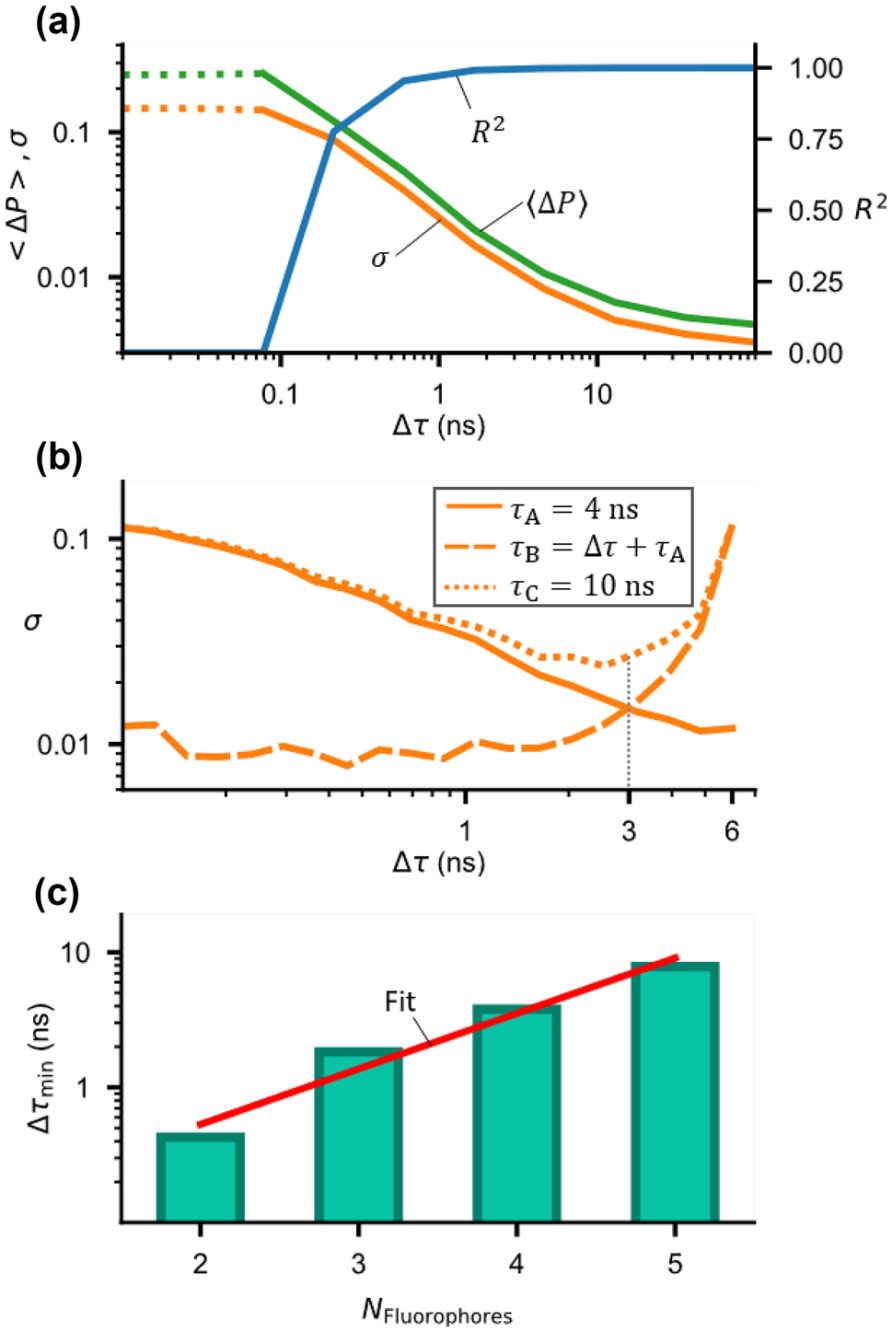
**a** Impact of the lifetime spacing on the accuracy (mean offset $\langle \Delta P\rangle$), the precision (standard deviation $\sigma$) and linear correlation (${R}^{2}$) using Monte-Carlo simulated data for a mixture of two fluorophores (${\tau }_{\mathrm{A}}=4 \, \mathrm{ns}, {\tau }_{\mathrm{B}}=\Delta \tau +{\tau }_{\mathrm{A}}$). **b** Standard deviation vs. lifetime spacing $\Delta \tau$ for a mixture three fluorophores. **c** Minimum equal spacing $\Delta {\tau }_{\mathrm{min}}$ for mixtures of up to five fluorophores at fixed standard deviation. The red line shows a linear fit (${R}^{2}=0.97$) of the log scaled results

For mixtures of two fluorophores, the mean offset (Fig. 3a, green curve) and standard deviation (Fig. 3a, orange curve) decrease with increasing spacing of the lifetimes $\Delta \tau$, while the linear correlation coefficient rapidly increases and saturates towards almost one (Fig. 3a, blue curve).

At low lifetime spacings $\Delta \tau$ (approx. below 0.08 ns), the linear correlation is zero (Fig. 3a, blue curve), while the mean offset (Fig. 3a, green dotted curve) and standard deviation (Fig. 3a, orange dotted curve) remains at values of approx. 25% and 15%, respectively. Since there is no linear correlation, it can be assumed that the determination of the fractional contributions is limited and is not possible when $\Delta \tau$ becomes too small. However, the ANN algorithms are trained to minimize the error of the determined fractional contributions, so that the ANN returns a result for $P$ that leads to a maximum value of the mean offset and standard deviation.

For mixtures of more than two fluorophores, the mean offset and the standard deviation depend on the spacing $\Delta \tau$ between all lifetimes. In order to demonstrate this, we determine the mean offset and standard deviation for a mixture of three fluorophores where two lifetimes were fixed (${\tau }_{\mathrm{A}}=4 \, \mathrm{ns}$ and ${\tau }_{\mathrm{C}}=10 \, \mathrm{ns}$ in accordance to the lifetimes for fluorescein and 2-amino-acridone) and the third lifetime was a variable ${\tau }_{\mathrm{B}}=\Delta \tau +{\tau }_{\mathrm{A}}$ (${\tau }_{\mathrm{A}}<{\tau }_{\mathrm{B}}<{\tau }_{\mathrm{C}}$) (Fig. 3b). Note, that for a clearer visualization, we only show the standard deviation (mean offset shows similar behavior, data not shown).

With increasing $\Delta \tau$, the standard deviation for ${\tau }_{\mathrm{A}}$ and ${\tau }_{\mathrm{B}}$ decreases, while it increases slightly for ${\tau }_{\mathrm{C}}$. This is to be expected since the spacing between ${\tau }_{\mathrm{A}}$ and ${\tau }_{\mathrm{B}}$ becomes larger and the spacing between ${\tau }_{\mathrm{B}}$ and ${\tau }_{\mathrm{C}}$ becomes smaller. When the spacing between ${\tau }_{\mathrm{A}}$ and ${\tau }_{\mathrm{B}}$ is larger than between ${\tau }_{\mathrm{B}}$ and ${\tau }_{\mathrm{C}}$, the standard deviation of ${\tau }_{\mathrm{B}}$ and ${\tau }_{\mathrm{C}}$ increases and that of ${\tau }_{\mathrm{A}}$ continues to decrease. Fig. 3b shows that at a lifetime spacing $\Delta \tau$ of about 3 ns, the standard deviations of ${\tau }_{\mathrm{A}}$ and ${\tau }_{\mathrm{C}}$ intersect and ${\tau }_{\mathrm{B}}$ is located at a minimum. This point corresponds to a uniform spacing between lifetimes.

Based on these findings, we determined the required minimal lifetime spacing $\Delta {\tau }_{\mathrm{min}}$ for a larger number of fluorophores at fixed standard deviation of 5% (Fig. 3c). With a larger number of fluorophores, $\Delta {\tau }_{\mathrm{min}}$ is also larger. A linear fit (${R}^{2}=0.97$) of the log scaled results yield

6 $$\Delta {\tau }_{min}={10}^{0.41\cdot {N}_{\mathrm{Fluorophores}}-1.1}.$$

When comparing the precision values from simulated (Fig. 3, $\sigma =1\%$ to 3 $\%$) to experimental data (Fig. 2e, $\sigma =7.5\%$ to $12.1\%$), the precision was slightly higher for the simulated data, although the simulated lifetimes were chosen in the same range as the experimental lifetimes. The difference is likely caused by uncertainties in the determination of the reference fractional contributions. It is worth noting that the experimental measurements and simulations were performed using a SPAD detector with a time resolution of 312.5 ps [6]. It is to be expected that with a higher time resolution even higher precisions could be obtained. The precision and accuracy also depend on the SNR of fluorescence lifetime data (see Suppl. Fig. 4) [35, 36]. By simulation, we could show that the precision and accuracy only slightly improve beyond a SNR > 100 that corresponds to the SNR in our experimental and simulated training data (see Suppl. Fig. 5).

Knowledge of the minimum lifetime spacing for a certain number of fluorophores that should be distinguishable in an experiment, allows the selection of the appropriate fluorophores and quantitative determination of their fractional contributions. Such multi-fluorophore discrimination is extremely interesting for applications such as the discrimination and quantitative determination of different biomolecules such as NADH/NAD^+^ [11], Ca^+2^ [37], turbulin, and lamin [38]. This allows monitoring of specific vital parameters such as the pH [39] of single cells [37, 38], or the in-vivo [8, 40–42] detection of cancerous tissue.

However, most fluorophores that are typically used have lifetimes of less than 20 ns [3, 7]. With these lifetimes, the number of distinguishable fluorophores is limited, e.g., mixtures of up to four fluorophores at an achievable standard deviation of 5%. Current research on fluorophores to achieve a wide range of different lifetimes will allow the number of differentiable fluorophores to be increased even further [43].

Previous studies have also shown that it is possible to distinguish mixtures with two or three fluorophores based on their lifetimes. These studies used conventional fit algorithms in which either the amplitude representative factors are determined together with the lifetimes [37, 40], or they are determined once individually in an unmixed state [38]. However, a quantitative comparison to our method is difficult, since those studies have not investigated the accuracy and precision of the determined fractional contributions.

As with most methods using ANN, the accuracy and precision of the determined values increases with the amount and the quality of data used for training [44]. Therefore, an increase in accuracy and precision with the proposed ANN-lifetime method can be expected, if more experimental data will be recorded. In perspective, this would also eliminate the initial training step with simulated data and the need to determine the lifetimes of fluorophores in a single experiment.

To summarize our study, we presented a new ANN-lifetime method for determining fractional contributions from lifetime measurements of fluorophore mixtures. With this approach fluorophores can be distinguished even when their emission spectra overlap. Thereby, the number of fluorophores is limited by the lifetimes’ differences, which highlights the enormous potential of the ANN-lifetime method for multi-fluorophore applications such as multiplex diagnostics or flow cytometry.

### Supplementary Information

Below is the link to the electronic supplementary material.

Supplementary file1 (DOCX 255 KB)

## Data Availability

Data underlying the results presented in this paper are not publicly available at this time but may be obtained from the authors upon reasonable request.
