# Supplementary material for: Artificial Neural Network (ANN)-Based Determination of Fractional Contributions from Mixed Fluorophores using Fluorescence Lifetime Measurements
Source: J Fluoresc. 2023 May 22;34(1):305–11. doi: 10.1007/s10895-023-03261-9 (PMC10808714; doi:10.1007/s10895-023-03261-9)
Supplement: Supplementary file 1 — Supplementary file1 (DOCX 255 KB) [file 10895_2023_3261_MOESM1_ESM.docx]

Supplementary Material

## Journal of Fluorescence

## Artificial neural network (ANN)-based determination of fractional contributions from mixed fluorophores using fluorescence lifetime measurements

Alexander Netaev^1 3^ ORCID: 0000-0002-6957-5270, Nicolas Schierbaum^1^ ORCID: 0000-0001-8977-5176, Karsten Seidl^1 2^ ORCID: 0000-0001-6197-5037

^1^ Fraunhofer Institute for Microelectronic Circuits and Systems, Finkenstr. 61, 47057 Duisburg, Germany

^2^ Department of Electronic Components and Circuits and Center for Nanointegration Duisburg-Essen (CENIDE), University Duisburg-Essen, 47057 Duisburg, Germany

^3^ E-mail: [alexander.netaev@ims.fraunhofer.de](mailto:alexander.netaev@ims.fraunhofer.de)

## Least Square (LS) Method with fixed Fluorescence Lifetimes


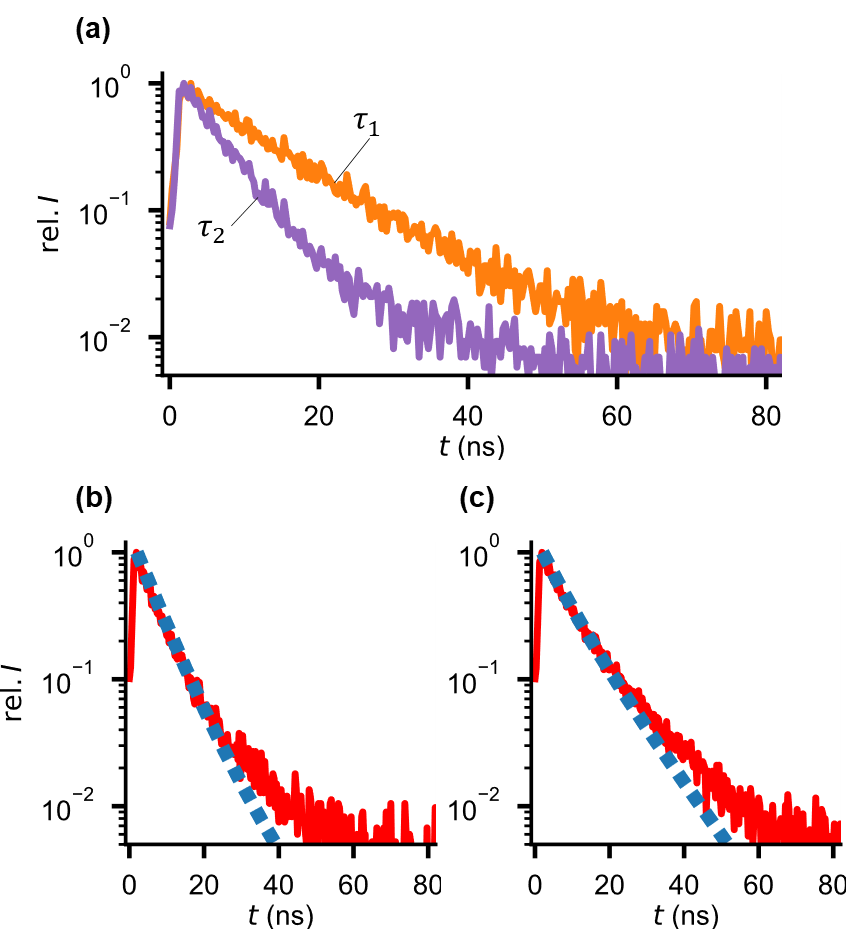


Fig. 1: Determination of fractional contributions in fluorescence lifetime measurements using non-linear LS method with the amplitude-representative factor as free fit parameter and the lifetime as constrained parameter. (a) Mono-exponential decay of fluorescence intensities of two separately measured fluorophores 2-amino acridone ($\tau_{1}=10.6 ns$) and acriflavine ($\tau_{2}=5.6 ns$). (b and c) Representative multiexponential decay of fluorescence intensities (red curves). The fractional contributions are in (b) $P_{Ref,1}=25\%; P_{Ref,2}=75\%$ and in (c) $P_{Ref,1}=28\%;P_{Ref,2}=72\%$. Corresponding fits based on LS method (blue dashed lines). The fit function in (b) and (c) are described by two amplitude-representative factors (b: $\alpha=0.13; \alpha_{2}=0.87; \chi^{2}=346$ and c: $\alpha_{1}=0.54; \alpha_{2}=0.46;\chi^{2}=111$), since the lifetimes are both constrained. The fractional contributions $P_{LS}$ are in b: $P_{LS,1}=22\%$; $P_{LS,2}=78\%$ and in c: $P_{LS,1}=69\%$; $P_{LS,2}=31\%$


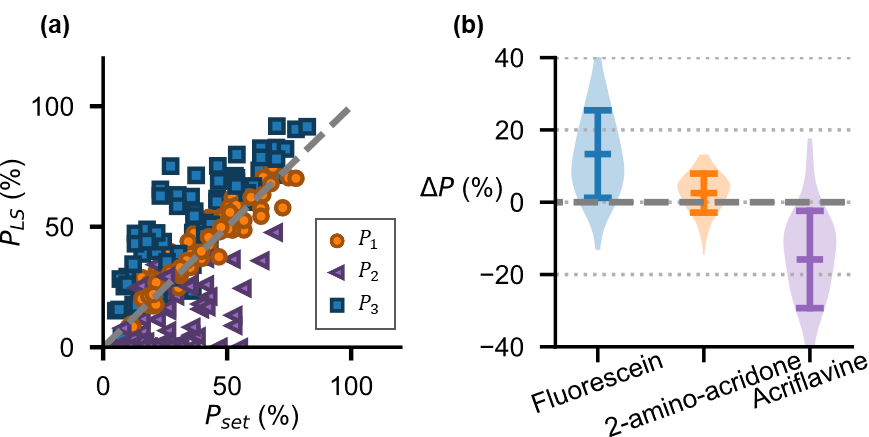


Fig. 2: Determination of fractional contributions by least square (LS) method. (a) $P_{LS}$ vs. $P_{Ref}$ show that the data points from the mixtures of three fluorophores with 2-amino-acridone (orange circles, $\tau_{1}=10.6 ns$), acriflavine (purple triangles, $\tau_{2}=5.6 ns$) and fluorescein (blue squares, $\tau_{3}=4.1 ns$), are linearly correlated ($R_{1}^{2}=0.89, R_{2}^{2}=0.23,R_{3}^{2}=0.7$). The gray dashed line corresponds to $P_{LS}=P_{Ref}$. (b) Violin plots show the distribution of offsets $\Delta P=P_{LS}-P_{Ref}$ for each fluorophore with respective mean value and standard deviation in mixtures of three fluorophores

## Training Datasets


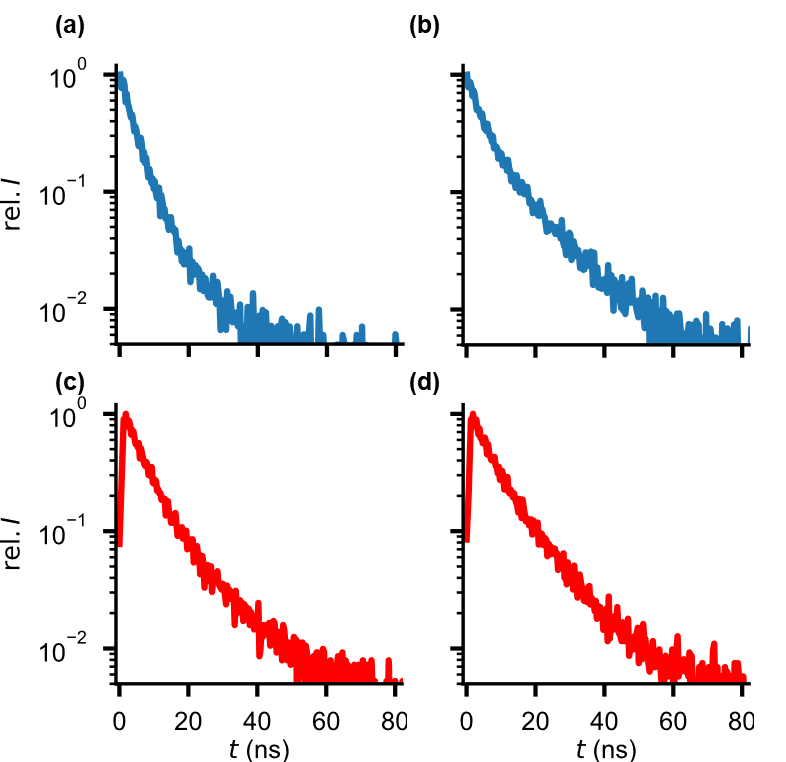


Fig. 3: Selections of ((a) and (b)) simulated and ((c) and (d)) experimental data used for training the ANN-lifetime algorithm

## Influence of Signal-to-Noise Ratio (SNR)


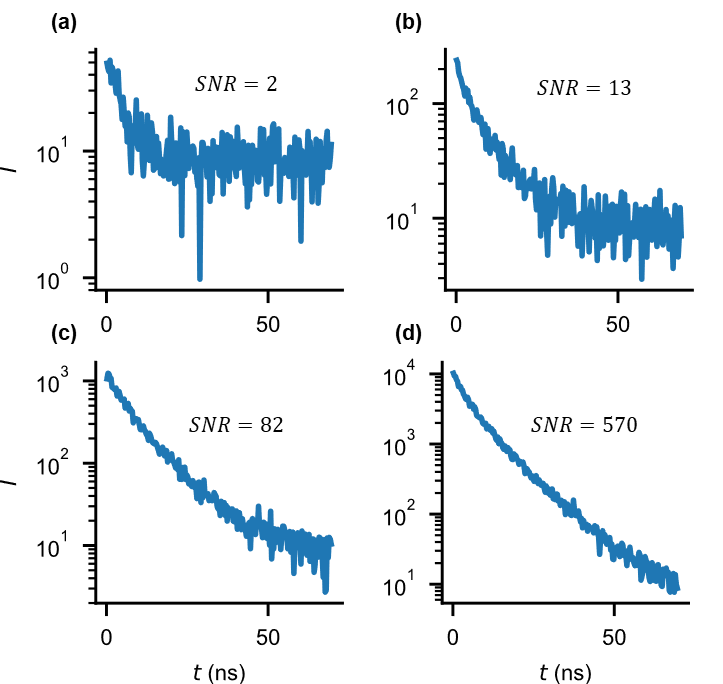


Fig. 4: Simulated fluorescence decay at different SNRs. The noise data is generated based on the Poisson distribution. The noise data was assumed to be uniformly distributed.


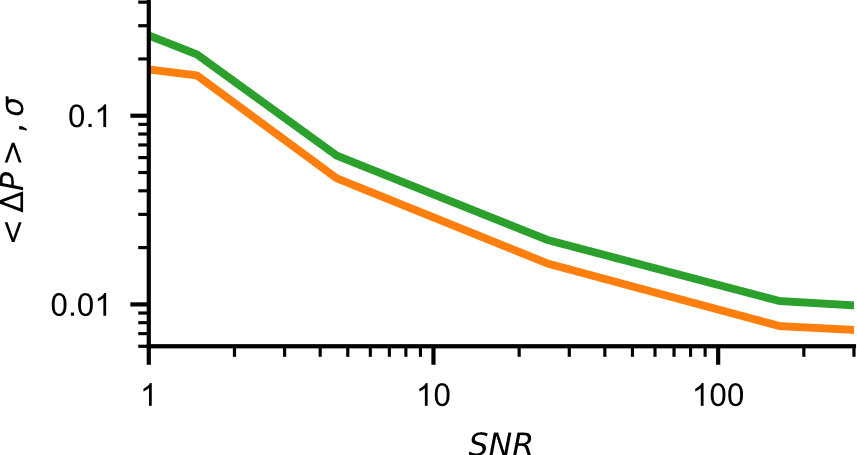


Fig. 5: Impact of the SNR on the precision (standard deviation $\sigma$) and accuracy (mean offset $\left\langle\Delta P \right\rangle$) using Monte-Carlo simulated data for a mixture of two fluorophores ($\tau_{A}=4 ns, \tau_{B}=10 ns$)
